# Sonochemically activated room temperature hydrosilylation of silicon nanoparticles †

**DOI:** 10.1039/d4na01067a

**Published:** 2025-04-04

**Authors:** Jonathan Trach, Shawna Williams, Brendan Michalczyk, Cole Butler, Alkiviathes Meldrum, John Washington, Jonathan G. C. Veinot

**Affiliations:** a University of Alberta 11227 Saskatchewan Drive Edmonton Alberta T6G 2G2 Canada jveinot@ualberta.ca; b Concordia University of Edmonton 7128 Ada Boulevard Edmonton Alberta T5B 4E4 Canada; c Department of Physics, University of Alberta Edmonton AB T6G 2E1 Canada

## Abstract

Hydrosilylation of terminal alkenes and alkynes on the surfaces of hydrogen-terminated silicon nanoparticles (H-SiNPs) has provided a convenient approach toward tailoring surface chemistry. These reactions have traditionally required thermal, photochemical, or chemical activation and are not necessarily compatible with all substrates and particle sizes. Herein, we demonstrate that hydrosilylation on silicon nanoparticles (Si NPs) can be promoted at room temperature by exposing the reaction mixture to a standard ultrasonic bath. This new approach provides surface coverages approaching 30% after 24 h. Introduction of traditional radical initiators to the reaction mixture followed by sonication reduced the reaction time by approximately 4-fold. The Si NPs functionalized using the presented sonochemical methods were compared with equivalent systems modified using conventional thermally- and radically-induced procedures and retain their appealing photoluminescent properties and were found to have slightly lower (*i.e.*, 27 *vs.* 33%), albeit comparable degrees of functionalization.

## Abstract 2

Silicon nanoparticles were functionalized using a standard benchtop ultrasonication bath, and the effectiveness of this sonochemical hydrosilylation method was explored in the context of reaction time, particle size, and activation conditions.

## Graphical abstract

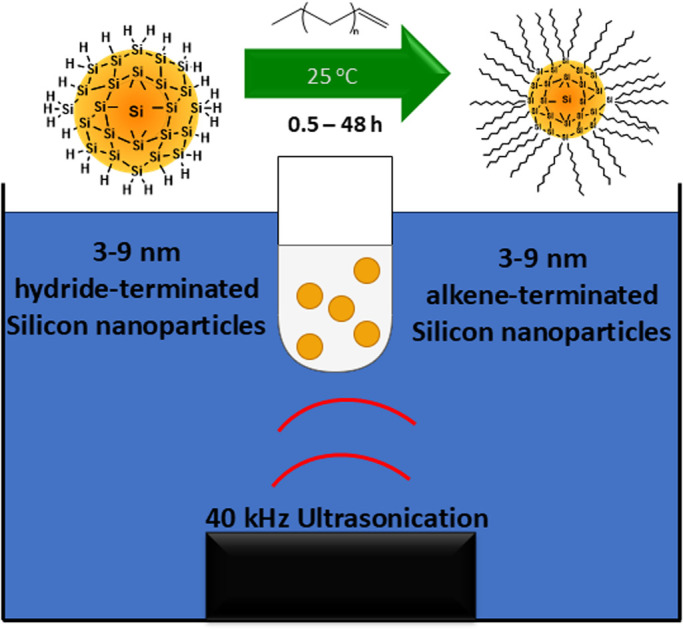

## Introduction

It is well-established that silicon is a foundational component of modern technological infrastructure.^1^ The biocompatibility and the inherent tunable properties (*e.g.*, photoluminescence) of nanoscale silicon^2–4^ have further cemented silicon as a versatile material that finds potential uses in a myriad of applications ranging from light-emitting diodes (LEDs),^5,6^ solar cells,^7,8^ sensors,^9–12^ drug delivery,^13,14^ and meta-materials,^15,16^ to quantum devices.^17,18^

Silicon is susceptible to oxidation under ambient conditions and, as a result, many of its favorable properties can be compromised if the oxidation is not mitigated – this is particularly true for nanoscale systems that possess high surface-area-to-volume ratios.^19^ Surface modification/functionalization is a common approach for imparting such stability and comes with the added benefits of solution processability, tunable luminescence, and tailorable surface reactivity.^20–23^ The most common approach toward modifying nanosilicon surfaces involves the reaction of terminal alkenes or alkynes with hydrogen-terminated surfaces. These so called hydrosilylation reactions formally add surface Si–H bonds across the carbon–carbon multiple bond of the substrate to form a robust covalent Si–C linkage. These reactions have been employed in organic chemistry for nearly a century;^24,25^ they have also been exploited in the modification of silicon surfaces for at least three decades.^26^ Hydrosilylation reactions on the surfaces of nanomaterials can be achieved using radical,^26^ thermal,^27^ and UV- and white-light initiation,^28,29^ as well as by using other specialized reagents (*e.g.*, Lewis acids, XeF_2_, PCl_5_, and precious metal catalysts).^30–33^ This diverse suite of hydrosilylation reactions provides substantial versatility and, through judicious selection of the reaction conditions, one can tune material properties such as surface coverage, surface packing (*i.e.*, monolayers *vs.* oligomers/polymers),^34^ and optical properties.^21^ It is informative to understand the corresponding limitations of each method.^35^

For radical-initiated reactions using AIBN (2,2′-azobis(2-methylpropionitrile)), the silicon nanoparticle functionalization reaction proceeds at 60–70 °C. This is markedly lower than the elevated temperatures required for thermal initiation (*ca.* 190 °C) and provides increased reagent compatibility (*e.g.*, access to low-boiling reagents). A disadvantage is that non-specific functionalization can be observed with alkenes (or alkynes) that possess an additional functional group.^35^ Generally, solvent compatibility (particle size dependent) is realized after 1–2 h and maximum surface coverage (*ca.* 30%) is achieved after *ca.* 16 h. Radical initiation also offers the advantage of providing monolayer functionalized surfaces, which leads to more readily processable particles and improved photoluminescence response (*i.e.*, higher quantum yield) compared to the higher temperature routes.^21,36^

High temperature (*i.e.*, 190 °C) thermally-activated hydrosilylation reactions are often the functionalization method of choice because they provide higher surface coverage. This reaction typically requires 24 h to complete and leads to the formation of oligomeric species on the particle surfaces. However, the high temperatures required are not compatible with low boiling, short chain alkenes. It is important to note the apparent higher degree of surface functionalization is the result of assumptions within the surface coverage estimates that rely on TGA (thermogravimetric analysis) as mass loss data is skewed high by the presence of surface bonded oligomers.^37^ Still, this approach is useful when a high surface density of functional groups is required for a given target application.^37^

For circumstances that require lower reaction temperatures (*e.g.*, introduction of short chain surface groups or temperature sensitive functionalities), surface hydrosilylation reactions can be initiated photochemically *via* exposure to UV or white light. This approach provides monolayer surface coverage of *ca.* 20% at room temperature after 3 h,^33^ however quantum confinement effects limit this option to comparatively small (<3 nm) nanoparticles and does not provide a route toward tailoring surface chemistry in larger systems.^34^ Functionalization of larger particles can be achieved using precious metal catalysts (*i.e.*, H_2_PtCl_6_, Wilkinson's Catalyst),^38,39^ however this brings with it contamination concerns that can compromise biological compatibility and optical response.^33,40^

Specialized reagents such as XeF_2_ or PCl_5_ also initiate surface functionalization upon reaction with alkenes and alkynes. These reactions are rapid (*i.e.*, 60 s to achieve completion) and offer substrate versatility along with substantially improved photoluminescence quantum yields.^31,41^ Despite their appeal, these specialized reagents introduce complications (*e.g.*, safe handling, cost, potential impurities) that limit scalability. To avoid the above complications, the development of new methods that offer size-independence, low temperature reactivity and a reasonable reaction rate are of interest.

Ultrasonication has previously been shown to activate hydrosilylation reactions on bulk silicon surfaces and offers an attractive option to initiate functionalization of H-SiNPs.^42^ Acoustic cavitation in an ultrasonic bath leads to the formation, growth, and implosive collapse of bubbles in liquids, producing micron-sized regions that can approach 5000 K and 1000 bar.^43–45^ It is reasonable that these localized conditions would activate hydrosilylation reactions. However, it is unclear from the earlier study involving bulk substrates if the nanoparticles will remain intact and/or if size dependent reactivity will be observed. Herein, we present a methodical investigation of sonochemically-initiated hydrosilylation on H-SiNPs.

## Materials

Sulfuric acid (reagent grade, 95–98%) was purchased from Caledon Laboratory Chemicals. Hydrofluoric acid (HF; electronics grade, 48–50%) was purchased from Fisher Scientific. Fuming sulfuric acid (reagent grade, 20% free SO_3_ bases), trichlorosilane (99%), toluene (HPLC grade), methanol (reagent grade), ethanol (100%, reagent grade), 2,2′-azobis(2-methyl propionitrile) (98%), 1-dodecene (95.0%), 1,2-dichlorobenzene (anhydrous, 99%), and benzene (anhydrous, 99.8%) were purchased from Sigma-Aldrich. A PureSolv purification system (Innovative Technology, Inc.) equipped with N_2_ as the operating gas was used to prepare dry toluene. Air- and water-free reagents (1-dodecene, methanol) were prepared *via* three freeze–pump–thaw cycles and addition of activated molecular sieves. All reagents and solvents were used as received unless otherwise specified.

### Preparation of hydrogen silsesquioxane

Hydrogen silsesquioxane (HSQ) was synthesized using a modified literature procedure.^46^ Briefly, a mixture of concentrated (70 mL) and fuming sulfuric acid (32.5 mL) was prepared in a three-neck round bottom flask purged with argon and equipped with an addition funnel and a Teflon coated stir bar. Dry toluene (210 mL) was then added to the acids *via* the addition funnel to obtain two layers. A mixture of dry toluene (510 mL) and trichlorosilane (75 mL) was prepared and subsequently added dropwise into the acid–toluene mixture. The toluene layer was isolated and washed with an aqueous sulfuric acid (33% v/v) solution. The organic layer was then dried over solid MgSO_4_ and CaCO_3_ for 12 h, followed by centrifugation at 11 000 rpm (12 739×*g*) and suction filtration to remove the excess solid. Finally, the solvent was removed using rotary evaporation and dried *in vacuo* to yield *ca.* 20 g of a white solid. The HSQ was stored under vacuum in the dark until use.

### Preparation of SiNP/SiO_2_ composite

Si NPs were prepared *via* thermal disproportionation of HSQ, using well-established procedures developed in our laboratory.^46^ HSQ (*ca.* 3 g) was heated in a yttria stabilized zirconia boat inside of a standard laboratory tube furnace (SentroTech, STT-1600C) to 1100, 1200, or 1300 °C under Ar flow to yield a composite comprising a silica matrix containing *ca.* 3, 6, and 9 nm Si inclusions, respectively. The composite was then mechanically ground using an agate mortar and pestle followed by shaking with glass beads in *ca.* 300 mL of ethanol using a standard wrist action shaker. Filtering and drying provided a fine brown powder.

### Preparation of hydride-terminated Si nanoparticles

The SiNP/SiO_2_ composite (*ca.* 500 mg) was dispersed in a 1 : 1 : 1 mixture of ethanol : distilled water : hydrofluoric acid (15 mL) in a PET beaker and stirred with a Teflon coated stir bar for *ca.* 1 h. The particles were extracted from the reaction mixture by dispersing in toluene (*ca.* 5 mL). The toluene extract was subsequently centrifuged at 3500 rpm (1300×*g*), the clear colourless toluene layer was decanted, and the orange pellet was washed twice more with fresh toluene (2 × 5 mL) and recovered each time upon centrifugation. These H-SiNPs were used immediately after purification.

### Sonochemical hydrosilylation procedure

In a typical reaction, a standard microwave reactor vial (Biotage Product 354833) was loaded with dry solvent (*i.e.*, toluene or 1,2-dichlorobenzene; *ca.* 4 mL) and 1-dodecene (*ca.* 1 mL) and sealed with a septum cap (Biotage Product 352298) inside of a N_2_ filled glovebox. The sealed vial was removed from the glovebox and a suspension of H-SiNPs (*ca.* 25 mg) in dry solvent (1 mL) was added *via* syringe transfer. For reactions involving solid radical initiators, the initiator (100 mg of 2,2′-azobis(2-methyl propionitrile) (AIBN) or 150 mg of benzoyl peroxide) was first dissolved in 1 mL of dry solvent before addition to the reaction vial *via* syringe. Chloroform, used as an initiator, was added (1 mL) directly *via* syringe to the sealed vial. The vial was then placed in a standard ultrasonic bath (Fisher Scientific, FS125) at 42 kHz for a predefined time (*i.e.*, 2, 8, 24 h). A water circulation system (Thermo Scientific, Neslab RTE 7) was used to maintain the water level and bath temperature at 25 °C throughout the reaction. The sealed reaction vial was then removed from the sonication bath and the contents directly transferred to a centrifuge tube. Methanol (*ca.* 5 mL) was added to the centrifuge tube to induce precipitation of the functionalized SiNPs and the resulting solid product was isolated *via* centrifugation at 11 000 rpm (12 739×*g*) for 20 minutes. The functionalized particles were then redispersed in dry toluene (*ca.* 1 mL) without sonication and then methanol (*ca.* 5 mL) was added to induce precipitation. The precipitate was then isolated *via* centrifugation at 11 000 rpm (12 739×*g*) for 20 minutes. This dispersion/centrifugation procedure was repeated twice. The purified functionalized particles (*ca.* 25 mg) were then dispersed without sonication in a minimum amount of benzene (*ca.* 1 mL) and freeze-dried before being characterized by FT-IR, TGA, XPS, and STEM.

### Fourier-transform infrared spectroscopy

Samples of hydrogen-terminated and functionalized SiNPs for FT-IR analyses were prepared by drop casting from dry toluene suspensions onto silicon wafers. The solvent was evaporated, and spectra were then acquired using a Thermo Nicolet 8700 FT-IR Spectrometer equipped with a microscope.

### Thermogravimetric analysis

Thermogravimetric analysis was obtained using a PerkinElmer Pyris 1 system in a flowing Ar atmosphere. Samples were prepared by loading functionalized SiNPs (*ca.* 2–5 mg) into a platinum pan and heating over a range of 20–700 °C, at a rate of 10 °C min^−1^. Surface coverage was estimated from TGA mass loss data as described in the ESI.† All reaction conditions were run with at least three replicates with a standard deviation of 1–7%, depending on particle size (see Table S5†).

### X-ray powder diffraction

Powder samples were placed on a zero-background Si(100) wafer and analyzed in a Bruker D8 Advance powder diffractometer, using a SSD160 detector and a Cu radiation source (*K*_α_1__ = 1.54056 Å), operating at 40 kV and 40 mA. Full-width at half-maximum values for Scherrer analysis were obtained by fitting the diffraction pattern peaks individually in OriginLab with a Gaussian peak shape.

### X-ray photoelectron spectroscopy

X-ray photoelectron spectroscopy (XPS) was performed using a Kratos Axis 165 Ultra X-ray Photoelectron Spectrometer equipped with a monochromatic Al Kα radiation source (1486.6 eV) operating at 210 W. High-resolution spectra were measured using an analyzer pass energy of 20 eV and a step of 0.1 eV. For survey spectra, a pass energy of 160 eV and a step of 0.5 eV were used. Spectra were calibrated to C 1s 284.8 eV using adventitious carbon. Sample preparation involved adhering solid SiNPs to carbon tape on a metal loading bar. Samples were analyzed and fit using CasaXPS, utilizing a Shirley-type background. The Si 2p region was fit using established values for the Si 2p_1/2_ and Si 2p_3/2_ spin–orbit coupling of 0.63 eV, with doublet area ratios fixed at 1 : 2 and full-width at half-maximum ratios fixed at 1 : 1.

### Scanning/transmission electron microscopy

Scanning/transmission electron microscopy (S/TEM) images were collected on a JEOL JEM-ARM200CF microscope with a Cold Field Emission Gun source. Images were collected with an accelerating voltage of 200 kV. High resolution (HR) TEM images were processed using Gatan Digital Micrograph software (Version 3.4.1). TEM samples were prepared by depositing a drop of a dilute toluene suspension of SiNPs onto a holey or ultra-thin carbon-coated copper grid (Electron Microscopy Inc.). The grid bearing the sample was kept in a vacuum chamber at a base pressure of 0.2 bar for at least 24 h prior to data collection. The particle size distribution was assembled as an average-shifted histogram as described by Buriak *et al.* for at least 300 particles.^47^ The sizes of individual particles were determined using ImageJ.

### Photoluminescence measurements

Photoluminescence (PL) spectra were acquired by exciting samples using the combined 352 and 364 nm lines of an argon ion laser and collecting the emission using an optical fiber connected to an Ocean Optics USB 2000+ Spectrometer. A 425 nm long-pass filter (LPF) was used to eliminate scattered light from the excitation source. The spectral response was calibrated using a blackbody radiator. Samples were prepared by dispersing the solids in toluene until the mixture was mostly clear (∼2 mg mL^−1^) and transferring ∼4 mL into a 1 cm × 1 cm quartz cuvette.

## Results and discussion

For the present study, SiNPs of predetermined sizes were prepared using established procedures involving thermal processing of HSQ. This approach affords silicon oxide composites containing well-defined Si(0) nano-inclusions that are readily freed from the oxide matrix by ethanolic hydrofluoric acid etching.^48^ The hydrogen-terminated surfaces of the resulting H-SiNPs were functionalized using a sonochemically-initiated approach. The reaction products were subsequently characterized to evaluate the nature of the functionalization (*i.e.*, surface group identity, degree of functionalization), integrity of the SiNP core, size dependence of the reaction, surface oxidation, and optical response.

**Fig. 1:**
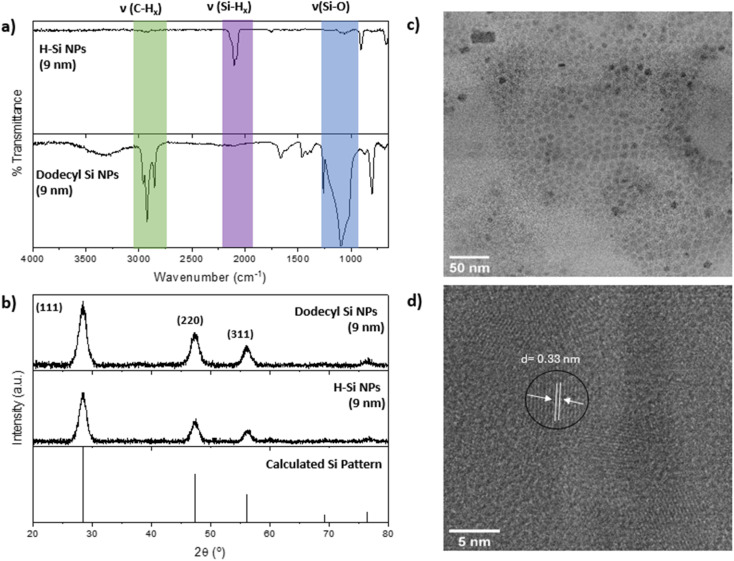
(a) FT-IR spectra of H-Si NPs (top) and dodecyl-terminated Si NPs (bottom). (b) XRD patterns of H-Si NPs, dodecyl-NPs, and calculated bulk Si. (c) Bright-field TEM image of dodecyl-Si NPs and (d) high-resolution image showing lattice fringes characteristic of Si(111).

As expected, for all parent particle sizes investigated, the H-SiNPs were orange amber solids incompatible with common organic solvents. The FT-IR spectrum of freshly etched 9 nm H-SiNPs (Fig. 1a) is representative of the other systems. It shows a feature characteristic of Si–H stretching at 2100 cm^−1^ as well as a comparatively weak Si–O–Si stretching feature at *ca*. 1000 cm^−1^ that arises because of limited oxidation resulting from ambient exposure during sample preparation. The poor solvent compatibility of H-SiNPs precludes effective sample preparation for electron microscopy and limits characterization using these methods. Hence, we estimate a crystallite size of 6.0 nm from the X-ray powder diffraction (Fig. 1b) using Scherrer analyses (Table S1†).^49^

Consistent with their incompatibility with organic solvents, the H-SiNPs form suspensions in toluene solutions of terminal alkenes (Fig. 5). Notably, sonication of these mixtures in a common bath sonicator afforded visually transparent mixtures after *ca.* 8 h. These observations are consistent with those previously observed for hydrosilylation reactions initiated using other means, and provide a qualitative evaluation of reaction progress.^50^ While it is tempting to assume the reaction is complete once visually apparent solvent compatibility of the SiNPs is achieved, previous studies involving functionalization of H-SiNPs showed maximum surface coverage required up to 24 h after initial observation of optical clearing of the reaction mixture.^37,50^

To elucidate the changes in surface chemistry resulting from sonochemical treatment of mixtures containing H-SiNPs in terminal alkenes, we first turn to FT-IR spectroscopy. For clarity the following discussion will focus on the observations made for the 9 nm diameter H-SiNPs/1-dodecene reaction system; equivalent representative data for other particle sizes (3 and 6 nm) and alkene chain lengths (octene and hexene) are presented in the ESI.† A straightforward comparison of the spectrum of H-SiNP with that obtained from the isolated product after sonicating in 1-dodecene for 48 h reveals evidence of alkyl functionalization (Fig. 1a). A new feature appears at *ca.* 2900 cm^−1^ that is characteristic of C–H stretching,^51,52^ with a corresponding diminished intensity in the Si–H stretching feature (*ca.* 2100 cm^−1^) and increased intensity of the Si–O–Si stretching feature (*ca.* 1000 cm^−1^). These spectral changes are consistent with hydrosilylation reactions proceeding on the SiNP surfaces and provide an explanation for the noted sonication-induced improvement in solvent compatibility.^36,53^

Despite the seemingly gentle nature of acoustic cavitation, sonication can induce extreme local conditions (*i.e.*, *ca.* 5000 K and 1000 bar)^43–45^ that can drive hydrosilylation reactions. However, these conditions could also possibly impact the local nanoparticle properties (*e.g.*, crystallinity). X-Ray Powder Diffraction (XRD) of the recovered product (Fig. 1b) shows reflections consistent with nanocrystalline silicon with a calculated crystallite diameter of 6.2 nm (Table S2†); this is in agreement with the equivalent evaluation of the parent H-SiNPs and is consistent with the integrity of the SiNPs being maintained. As XRD only probes the bulk nature of a sample, we turn to electron microscopy for a more direct measure of particle size and insight into the effects of prolonged sonication on local crystallinity. The solvent processability imparted by sonochemically-induced functionalization facilitates analysis of particles using bright-field STEM and HRTEM. Fig. 1c and d show representative images of functionalized SiNPs (*d* ∼9 nm) recovered from a typical 48 h exposure to ultrasonication in 1-dodecene. These representative images are equivalent to what is commonly observed for SiNPs functionalized using other hydrosilylation methods, and we can thus conclude that local crystallinity remains.^4,20,31^

**Fig. 2:**
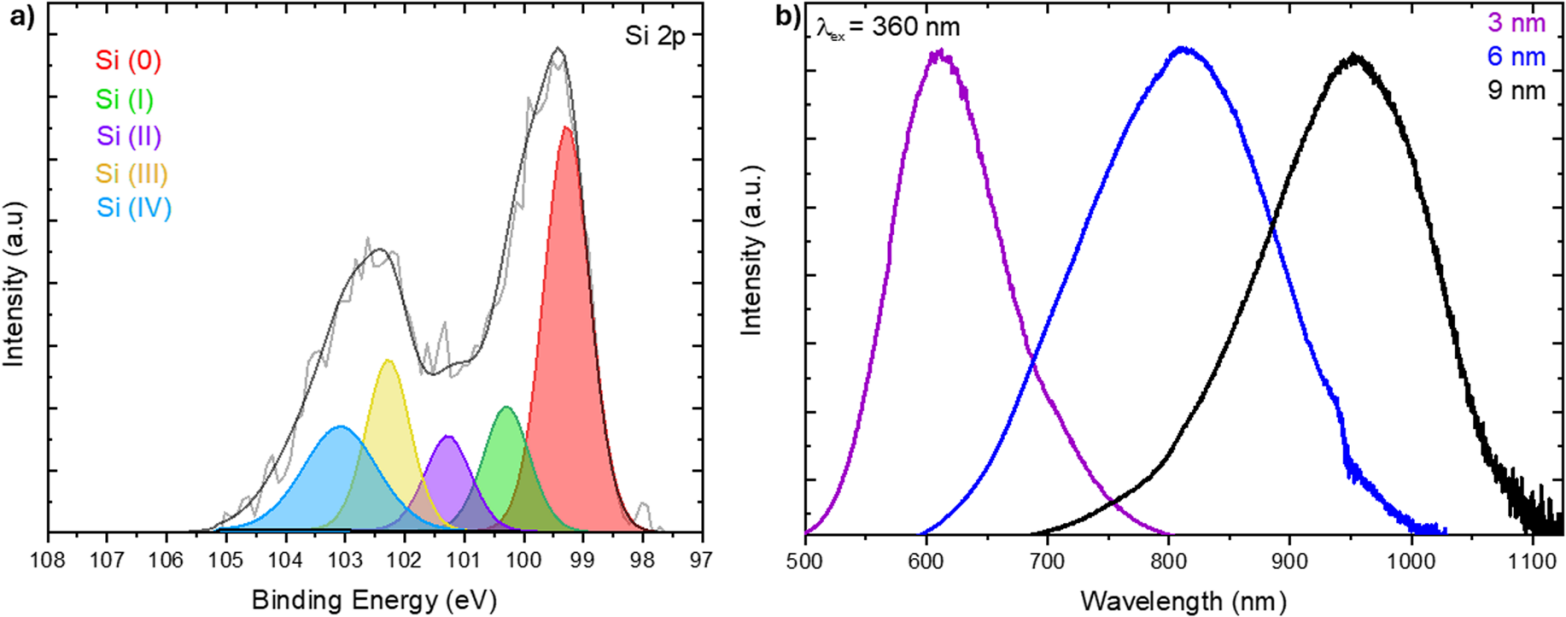
(a) High resolution Si 2p XP spectrum of 9 nm dodecyl SiNPs sonicated for 24 h. Si 2p_1/2_ envelopes omitted for clarity. (b) Normalized photoluminescence spectra of 3 (purple), 6 (blue), and 9 (black) nm dodecyl-SiNPs sonicated for 24 h.

Knowing that the composition and the corresponding oxidation states of the constituent elements can influence the properties of SiNPs, we investigated the present systems using XPS. The survey XP spectrum (Fig. S3†) confirms that the present SiNPs are comprised of only Si, C and O with evidence of trace (*ca.* 1%) F.^54^ A representative high-resolution spectrum of the Si 2p region obtained from sonication of H–Si NPs with 1-dodecene is presented in Fig. 2a. Fitting the spectral envelope to the standard silicon oxidation states reveals a dominant Si(0) contribution (43 atomic%, centered at 99.6 eV), with smaller components arising from Si(i) (13 atomic%, centered on 100.6 eV), Si(ii) (9 atomic%, centered on 101.6 eV), Si(iii) (17 atomic%, centered on 102.6 eV) and Si(iv) (17 atomic%, centered on 103.4 eV).^55^ These observations are consistent with the FT-IR analyses noted above and suggest dodecyl-terminated SiNPs prepared *via* the present sonication method bear similar surface oxidation to that which is observed for equivalent dodecyl-SiNPs prepared *via* standard radical-initiated reactions.^50^

Another useful metric for qualitatively evaluating silicon nanoparticle integrity is photoluminescence response (Fig. 2b). The as-prepared dodecyl-SiNPs exhibited the expected size dependent photoluminescence, with small particles (3 nm) emitting orange light (*λ*_max_ = 612 nm) upon UV exposure; an increase in size resulted in a red-shift of the emission wavelength (*λ*_max_ = 812 nm and 953 nm for 6- and 9-nm SiNPs, respectively). The spectra presented are consistent with conventionally (*e.g.*, radical-initiated, thermal) functionalized SiNPs, and show that sonochemical treatment does not compromise the SiNP photoluminescence response.^21,36^

**Fig. 3:**
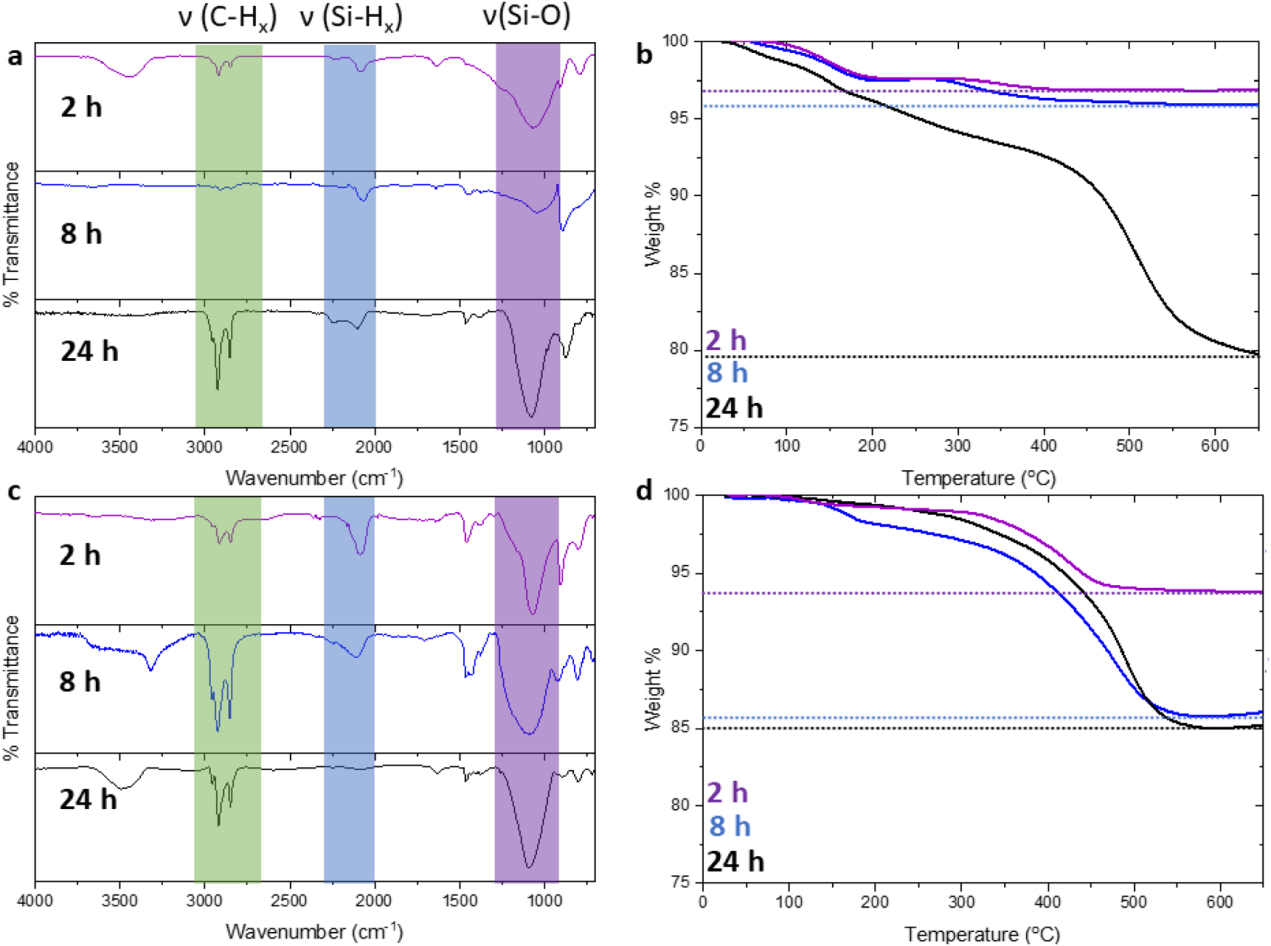
(a) FT-IR spectra of dodecyl-terminated Si NPs obtained from sonochemical reactions in the absence of a radical initiator for 2 h (purple), 8 h (blue), and 24 h (black) with (b) corresponding TGA data. (c) FT-IR spectra of dodecyl-terminated Si NPs recovered from reactions involving AIBN for 2 h (purple), 8 h (blue), and 24 h (black) with (d) corresponding TGA data.

Having demonstrated that sonochemical treatment of H-SiNPs in the presence of terminal alkenes induces surface hydrosilylation, the next step is to investigate the influence of various reaction parameters (*i.e.*, reaction time, particle size) on the nature of the resulting product. The first parameter investigated was reaction time. Reaction mixtures of 9 nm H-SiNPs and 1-dodecene were prepared in parallel and sonicated for 2, 8, and 24 h. The freeze-dried particles were recovered and the degree of functionalization was evaluated using thermogravimetric analysis (Fig. 3b) to estimate surface coverage. Generally, we observe that surface coverage increases through 3, 4, and 27% for 2, 8, and 24 h reaction times, respectively. This trend is also reflected in the corresponding intensities of the Si–H (*ca.* 2100 cm^−1^) and C–H (*ca.* 2900 cm^−1^) stretching features in the respective FT-IR spectra (Fig. 3a). Thus, longer reaction times lead to higher degrees of functionalization.

In an effort to increase the surface coverage, shorten reaction times, and gain insight into the reaction mechanism, the effects of adding common radical initiators (*e.g.*, AIBN, benzoyl peroxide, chloroform) to the reaction mixture were probed. Fig. 3c shows the FT-IR spectra of the products obtained from the sonication reaction of H-SiNPs with 1-dodecene in the presence of AIBN for the indicated times. As was the case for initiator-free reactions, when initiators are introduced to the reaction mixture we noted a progressive decrease in the intensity of the Si–H stretching feature at 2100 cm^−1^ with reaction time. In fact, after 24 h the Si–H feature decreases to the point that it is barely baseline resolved. Looking to the corresponding intensity of the C–H_*x*_ stretching feature (*ca.* 2900 cm^−1^) that arises from attachment of an alkyl moiety, we note that it initially increases with reaction time (*i.e.*, from 2 to 8 h) and then remains constant within the sensitivity of the measurement upon increasing the reaction time to 24 h. While the intensity of the C–H_*x*_ stretching feature does not increase with reaction time after 8 h, the intensity of the Si–O feature at *ca.* 1100 cm^−1^ does. These observations are consistent with surface oxidation occurring after prolonged sonication and a corresponding loss of Si–H. The intensity of the Si–O feature does not increase between 2 and 8 h of reaction time. These observations are consistent with the degree of surface functionalization reaching a maximum after *ca.* 8 h which is also reflected in our TGA analyses that reveal surface coverages of 7, 17, and 18% for AIBN initiated reactions after 2, 8, and 24 h, respectively (Fig. 3d). The impact of including a radical initiator in the reaction mixture is most prominent for short (*i.e.*, 2 h) duration reaction times. Fig. S2† shows the qualitative change in the reaction mixture appearance when a radical initiator (AIBN) is included – surface functionalization proceeds more rapidly with the reaction mixture becoming transparent after 1–2 h. TGA also reveals that surface functionalization nears a limiting value (*i.e.*, *ca.* 18% surface coverage) after only 8 h. This is in stark contrast to reactions without radical initiators that approach transparency after 8 h and maximum surface coverage (*i.e.*, *ca.* 27%) after 24 h.

These observations are also reflected in the corresponding FT-IR spectra – comparing the C–H_*x*_ stretching band at *ca.* 2900 cm^−1^, the intensity of this feature is stronger when a radical initiator is present (Fig. 3a *vs.* c). Although introduction of a radical initiator does not provide an increase in maximum surface coverage, the present sonication approach achieves coverages approaching what has been reported previously for standard radical-initiated reactions.^50^

We speculate that the noted difference in surface coverage in products involving 1-alkenes of different chain lengths (Fig. S12†) arises because of the limited solvent compatibility of the H-SiNPs. The initial reaction mixtures are heterogeneous – H-SiNPs are not “soluble” in the solvent mixture and are present as agglomerates. As the reaction proceeds and SiNPs at the agglomerate surfaces are partially functionalized, they become compatible with the reaction mixture. This results in the SiNP aggregates breaking up and the reaction mixture eventually appearing visually transparent, however small aggregates remain. When the reaction involves comparatively short 1-alkenes, surface functionalization affords less effective steric stabilization of the particles than when longer 1-alkenes are employed. As a result, the SiNPs functionalized with short chain alkenes are less compatible with the reaction mixture than their long-chain counterparts and are less available to participate in surface hydrosilylation reactions. This limits their reactivity and surface coverage. These observations are equivalent to what has been reported previously for radical-induced hydrosilylation.^50^

**Fig. 4:**
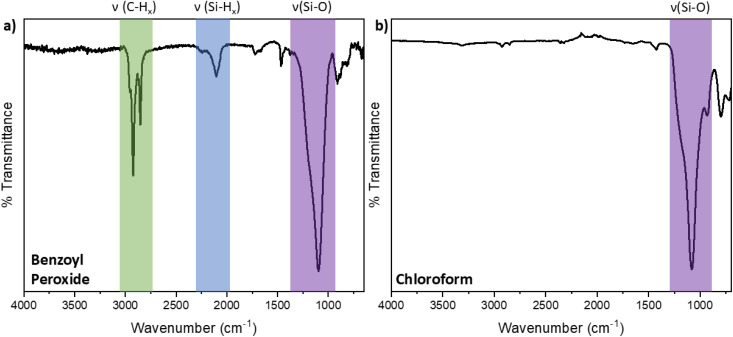
FT-IR Spectra of 9 nm dodecyl-functionalized SiNPs sonicated for 24 h with (a) benzoyl peroxide and (b) chloroform.

In addition to the radical reactions involving AIBN, we also explored using other initiators to probe the generality of the sonication method. Benzoyl peroxide is a peroxide-based initiator that exhibits similar reactivity to AIBN,^37^ and chloroform was recently demonstrated to activate hydrosilylation on the surfaces of small (*i.e.*, *d* = 3 nm) SiNPs at room temperature.^56^ It has been proposed that the later reaction proceeds *via* the decomposition of chloroform into phosgene and hydrochloric acid to produce radicals.^56^ To compare reactivities, equivalent reactions involving 9 nm H-SiNPs and 1-dodecene with benzoyl peroxide and chloroform radical initiators were performed. Benzoyl peroxide-initiated reactions provided equivalent products to those obtained from AIBN (Fig. 4a, 18% surface coverage, see Fig. S5†) while reactions involving chloroform provided no appreciable functionalization as evidenced by the lack of C–H stretching features in the FT-IR analysis (Fig. 4b).

As the previous literature study was confined to functionalization of small H-SiNPs, we attempted sonochemical functionalization of 3 nm H-SiNPs in the presence of chloroform. After only 2 h the reaction mixture cleared, and the FT-IR spectrum was consistent with minimal functionalization and TGA results (Fig. S5†) showed 3.8% surface passivation. This is comparable to the *ca.* 3% coverage obtained from reactions run without initiator and suggests that sonication does not promote radical formation in chloroform. To examine whether the solvent impacts the hydrosilylation efficiency, reactions were performed in 1,2-dichlorobenzene. These yielded comparable results to experiments conducted in toluene (surface coverage of *ca.* 7% without initiator, and *ca.* 40% with initiator – see Fig. S6†) and suggest that under the presented conditions, while recognizing the limited scope of solvents compatible with SiNPs, that solvent media has limited impact on the progression of surface hydrosilylation of terminal alkenes.

### Dependence of reactivity on H-SiNP size

Having noted an apparent size dependence of sonochemical functionalization initiated by chloroform we explored similar effects with other initiators (*i.e.*, AIBN, benzoyl peroxide) by repeating the sonochemical reactions noted above with 3 and 6 nm diameter H-SiNPs. Fig. S7† shows TGA plots for the products of sonochemically activated reactions involving 3, 6, and 9 nm diameter H-SiNPs, 1-dodecene, and AIBN for 2 and 24 h. The data reveals some limited size dependence; a 24 h reaction involving 6 nm H-SiNPs gave the highest surface coverage (35%), followed by 3 nm (22%), and finally 9 nm (18%) H-SiNPs. This same trend holds true for all functionalization times investigated and is also observed for reactions without radical initiators. While the TGA plots show a straightforward increase in total mass loss with decreasing particle size, calculated surface coverages (accounting for the differing amount of surface sites over different particle sizes) in Table S5† show that the 6 nm particles have the highest degree of functionalization.

**Fig. 5:**
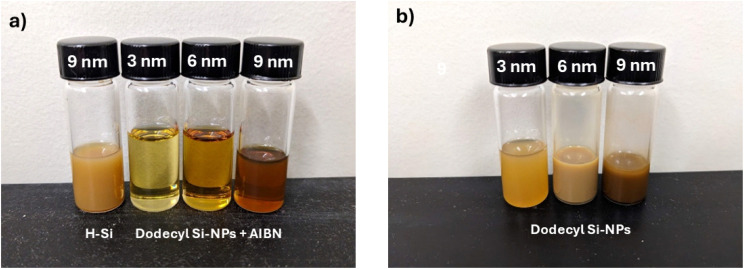
Qualitative appearance of toluene suspensions containing unfunctionalized Si–H and functionalized dodecyl Si-NPs of indicated sizes for reactions after 2 h of sonication (a) with and (b) without AIBN radical initiator.

Fig. 5 shows photographs of toluene suspensions of 1-dodecyl functionalized Si NPs of different sizes. After just 2 h of sonication, the reaction mixtures containing radical initiators (Fig. 5a) have become optically transparent for all particle sizes. This is in contrast to the solutions without initiator (Fig. 5b), all of which maintained their cloudy appearance. The appearance of these transparent mixtures qualitatively supports the proposal that, across all particle sizes, the increased rate of functionalization with AIBN present remains in effect.

**Fig. 6:**
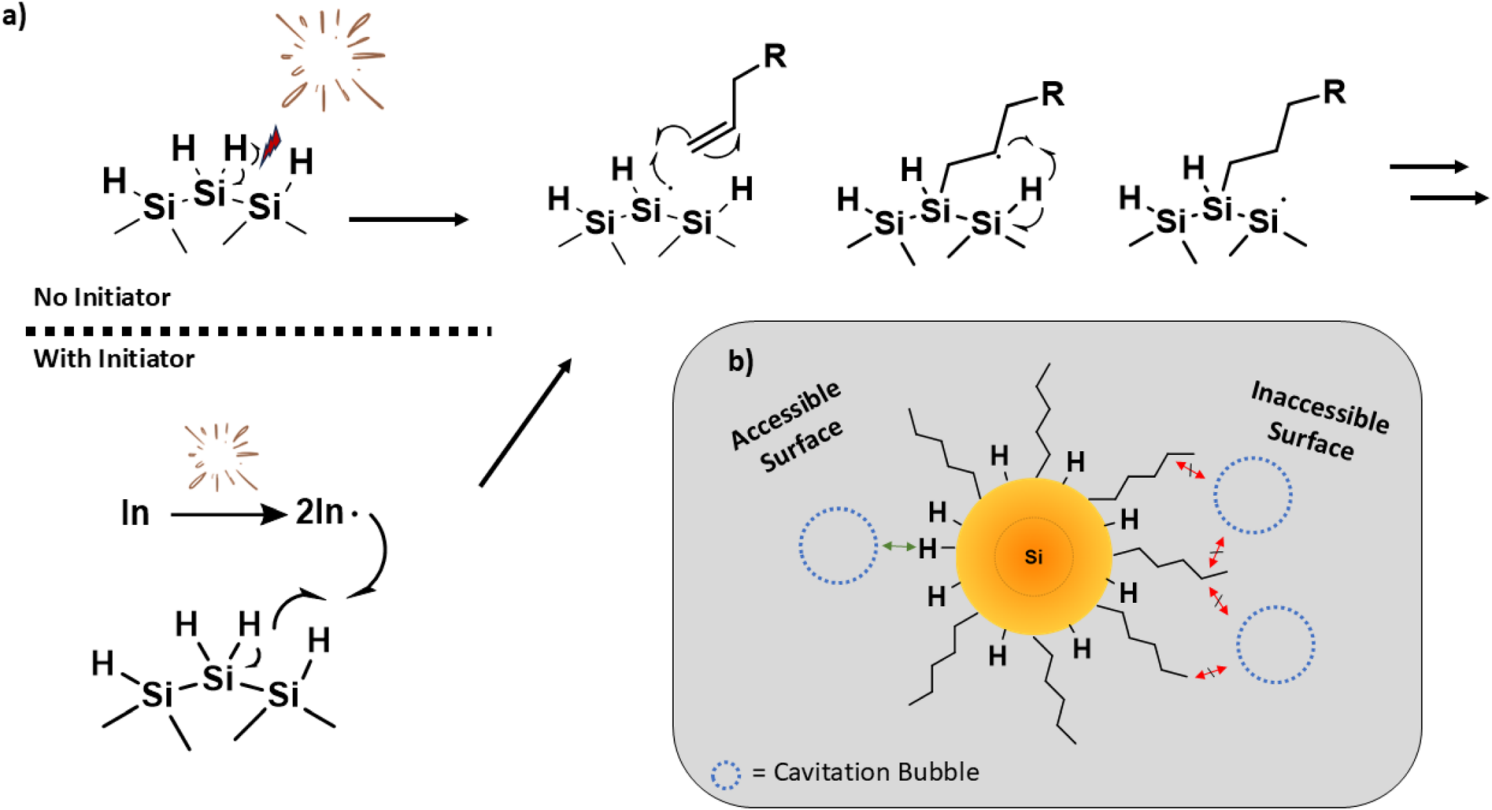
(a) A cavitation bubble collapsing near a H-SiNP particle, breaking a surface Si–H bond or activating a radical initiator, which then activates the SiNP surface to alkene hydrosilylation. (b) Cavitation bubble unable to break SiNP surface bonds due to reduced access from alkene blockage.

Having found that ultrasonication induces surface reactivity comparable to conventional approaches with H-SiNPs, it is useful to consider the mechanism by which the reaction may proceed. A proposed reaction mechanism analogous to the broadly accepted thermal- and radical-initiated hydrosilylation reactions on nanoscale and bulk silicon surfaces is illustrated in Fig. 6a and b, respectively.^57–59^ The collapse of cavitation bubbles created during ultrasonication creates localized regions of intense heat and pressure that can break bonds (*e.g.*, Si–Si, Si–H) at the SiNP surface.^60,61^ For convenience Fig. 6a only illustrates homolytic cleavage of surface Si–H bonds, however similar cleavage of Si–Si bonds cannot be neglected given the relative bond energies (*i.e.*, Si–H = *ca.* 339 kJ mol; Si–Si = *ca.* 226 kJ mol^−1^).^60^ The resulting surface radical reacts with a terminal alkene in close proximity, yielding a robust Si–C bond and a secondary radical at the C2 position of the surface tethered alkyl chain. The secondary radical then abstracts an adjacent H˙ and re-generates a surface radical. This process repeats, resulting in propagation of the radical reaction on the SiNP surface. We propose that the addition of a radical initiator increases the likelihood of radical formation (*via* the initiator providing more radicals to activate the Si surface), and thus speeds the reaction (Fig. 6a).

Considering this mechanism, if the sonochemically activated reaction is to proceed, it is necessary for the cavitation bubbles to collapse at or near an unfunctionalized region of the SiNP surface; if the density of surface groups on the SiNP is suitably high, the influence of the cavitation bubble collapse is lost and the reaction will stop (Fig. 6b). Consequently, the hydrosilylation reaction will not proceed past a certain threshold of surface coverage, and will slow as the reaction proceeds.

We have previously demonstrated that SiNPs prepared *via* thermal disproportionation of HSQ exhibit a size dependent core shell structure.^62^ The smallest nanoparticles SiNPs (*i.e.*, *d* ∼3 nm) are dominated by disordered surface atoms, *d* ∼6 nm SiNPs possess a quasi-ordered core and disordered surface, and *d* ∼9 nm SiNPs have a crystalline core, quasi-ordered subsurface and disordered surface. In this context, the smaller SiNPs possess a higher relative concentration of species such as Si–H_*x*_ that are susceptible to homolytic cleavage induced by sonication.^57^ This could lead to the higher surface coverage observed for the *d* = 3 and 6 nm SiNPs compared to their 9 nm counterparts.

The present SiNP systems provide a unique opportunity to investigate the role of surface disorder on the degree of functionalization. Over-etching of *d* ∼9 nm H-SiNPs allows removal of much of the disordered surface while decreasing the overall particle size.^36^ For the present study, over-etching provided H-SiNPs with dimensions of *d* ∼6 nm, which have a more ordered surface compared to normally prepared 6 nm Si NPs. The over-etched particles were sonicated with 1-dodecene for 24 h, and gave reduced reactivity in hydrosilylation, as indicated by the TGA data in Fig. S8.† The average surface coverage of the overetched particles is 14%, which is significantly lower than reactions undertaken with traditionally etched 6 nm particles (*i.e.*, *ca.* 44%).

## Conclusions

Hydride-terminated silicon nanoparticles, when subjected to a straightforward sonication method in the presence of 1-dodecene show successful functionalization. The addition of radical initiators such as AIBN and benzoyl peroxide results in improved surface coverage. The extent of this hydrosilylation was investigated using FT-IR and TGA analysis and shown to produce surface coverages analogous to conventional thermal methods (*ca.* 35%). However, and of note, the sonication reactions were all conducted at room temperature. The effects of reaction time and particle size were also investigated. The presence of radical initiator speeds up the reaction time by four-fold, but the reaction proceeds with or without initiator within 24 h. There was a slight size dependence observed, with smaller particles showing higher degrees of functionalization. A similar mechanism to conventional hydrosilylation is proposed, wherein the collapse of cavitation bubbles leads to silyl radicals on the particle surface. This approach could lead to hydrosilylation being more accessible with sensitive reagents and could also allow more control over the surface when using alkenes with additional functional groups.

## Data availability

The data supporting the findings of this report have been included in the article and the ESI,† with raw data available upon request of the corresponding author.

## Conflicts of interest

There are no conflicts of interest to declare.

## Supplementary Material

NA-007-D4NA01067A-s001

NA-007-D4NA01067A-s002
