# Supplementary material for: Sonochemically activated room temperature hydrosilylation of silicon nanoparticles
Source: Nanoscale Adv. 2025 Apr 4;7(10):3018–27. doi: 10.1039/d4na01067a (PMC11969236; doi:10.1039/d4na01067a)
Supplement: NA-007-D4NA01067A-s002 [file NA-007-D4NA01067A-s002.pdf]

Supplementary Information (SI) for Nanoscale Advances.
This journal is © The Royal Society of Chemistry 2025

Supporting Information for:

Sonochemically Activated Room Temperature Hydrosilylation of Silicon Nanoparticles

Jonathan Trach[1], Shawna Williams[1], Brendan Michalczyk[2], Cole Butler[1], Alkiviathes Meldrum[3], John Washington[2], and Jonathan G. C. Veinot[*1]

[1] Department of Chemistry, University of Alberta, 11227 Saskatchewan Drive, Edmonton, Alberta T6G 2G2, Canada

[2] Concordia University of Edmonton, 7128 Ada Boulevard, Edmonton, Alberta T5B 4E4, Canada

[3] Department of Physics, University of Alberta, Edmonton, AB T6G 2E1, Canada

Table of Contents

Surface Coverage Calculations …………………………………………………………… 4-5

Tables ……………………………………………………………………………………… 6-8

Table S1. Scherrer Analysis for 9 nm hydride-terminated particles …………… 6

Table S2. Scherrer Analysis for 9 nm dodecyl-terminated particles ……….…. 6

Table S3. Scherrer Analysis for 6 nm dodecyl-terminated particles ……….…. 6

Table S4. Scherrer Analysis for 3 nm dodecyl-terminated particles …..……… 7

Table S5. Calculated Surface Coverage of SiNPs Averaged Over 55 Radical-Initiated Reactions ..………………………………………………………….…………8

Table S6. Weight Loss and Surface Coverage Values for TGA data presented………………………………………………………………………………9

Figures ………………………………………………………………………………10-22

Figure S1. (a) Bright-Field TEM Image of 6 nm dodecyl-SiNPs and (c) Dark-Field TEM Image of 3 nm dodecyl-Si NPs, with corresponding average shifted histogram size distributions (b) and (d). e) Average shifted histogram for 9 nm particles, corresponding to Figure 1c.……………………………………………….. 10

Figure S2. A 15x timelapse of the sonochemical hydrosilylation of 9 nm Si NPs with AIBN and 1-dodecene, showing the transition from opaque orange/brown to a transparent dark red solution ………………………………………………….. 11

Figure S3. Survey XP Spectrum of 9 nm Si NPs sonicated with 1-dodecene for 24 h……………………………………………………………………………………...12

Figure S4. TGA Plot of Si NPs functionalized with 1-dodecene and AIBN at 65 ^{o}C (blue) and at 195 ^{o}C with no initiator (red) after 24 h.…………………………….. 13

Figure S5. a) FTIR Spectra and b) TGA data of Benzoyl Peroxide initiated hydrosilylation (blue) and chloroform initiated hydrosilylation (black) using 1-dodecene and 3 nm SiNPs……………………………………………………………14

Figure S6. a) FTIR Spectra and b) TGA data of 6 nm Si NPs sonicated with 1-dodecene in 1,2-dichlorobenzene for 8 h, both in the presence and absence of AIBN…………………………………………………………………………………15

Figure S7. TGA plots of 3 (purple), 6 (blue) and 9 (black) nm Si NPs sonicated with Dodecene and AIBN for a) 2 h and b) 24 h……………………………………16

Figure S8. TGA plot of ca. 6 nm over-etched Si NPs (black) and traditionally etched 6 nm Si NPs (blue) sonicated with Dodecene for 24 h ..…………………17

Figure S9. XP Spectra of a) 3 and b) 6 nm dodecyl-SiNPs sonicated for 24 h with AIBN, with corresponding survey spectra in c) and d)…………………………..…18

Figure S10: XP Spectra of H-SiNPs which are 3 (a), 6 (c), and 9 (e) nm in size, along with corresponding survey spectra (b, d, and f)……………………………19

Figure S11: a) Photoluminescence spectra of 3, 6, and 9 nm H-SiNPs and b) photograph comparing photoluminescence response of H-SiNPs and Dodecyl SiNPs……………………………………………………………………………….….20

Figure S12. XRD Patterns obtained from 3 and 6 nm dodecyl-SiNPs sonicated for 24 h with AIBN……………………………………………………………………..21

Figure S13. TGA Plot of sonochemically-functionalized Si NPs for 24 h obtained from 24 h reactions with AIBN and 1-hexene, 1-octene, or 1-dodecene, to yield surface coverages of 11, 15, and 21 %, respectively.……………………………. 22

Surface Coverage

Thermal Gravimetric Analysis (TGA) was used to characterize the degree of functionalization of the Si NPs described. The output of this analysis technique is a percentage of the total mass loss over a temperature range, which is independent of particle size, surface characteristics, or ligand used. To properly compare the extent of functionalization between different nanoparticle sizes, this mass loss must be converted to surface coverage, which was done with the following procedure:

1. The amount of Si atoms present were calculated, expressed in the number of Si atoms present per nm, utilizing the density and atomic mass of Si, as well as Avogadro's number
2. Assuming a 'surface radius' that is one Si atom thick, the number of atoms that are on the surface of the particle were calculated, and assuming the surface Si atoms average out to being SiH_2, the number of available surface sites to react with are twice the number of surface atoms.
3. Based on the ligand used (e.g. dodecene), the molecules that were lost in the course of TGA were calculated using the mass loss and the molar mass of the ligand
4. The theoretical number of silicon particles that would be present in the analyzed sample can be determined from the Si/nm^3 from (1), with an additional option for tuning the accuracy of the calculation – the surface of the SI NPs are likely to be somewhat oxidized, and thus not all SiH_x, so some amount of SiO_2 can be accounted for by using a corrected molar mass term via a weighted average calculation.
5. The surface coverage can then be calculated from the molecules of ligand lost per Si NP (step 3 / step 4), and the number of reactive sites (step 2).

$$\frac{\#\, Si\ Atoms}{nm^3} = \frac{Density\left(\frac{g}{nm^3}\right)}{Molar\ Mass\left(\frac{g}{mol}\right)} * N_A\left(\frac{atoms}{mol}\right) \quad (1)$$

$$\#\, Surface\ Reactive\ Sites = \frac{4}{3}\pi\left(r^3 - \left(r - r_{Si}\right)^3\right) * \frac{\#\, Si\ Atoms}{nm^3} * 2 \quad (2)$$

$$Molecules\ of\ Ligand\ Lost = \frac{Mass\ Loss\ (g)}{Molar\ Mass\left(\frac{g}{mol}\right)} * N_A \quad (3)$$

$$Theoretical\ Particle\ Count = \frac{\frac{\#\, Si\ Atoms}{nm^3}}{N_A} * V_{Particle} * Molar\ Mass_{corr.} \quad (4)$$

$$\%\ Surface\ Coverage = \frac{\frac{Molecules\ of\ Ligand\ Lost}{Theoretical\ Particle\ Count}}{\#\, Surface\ Reactive\ Sites} \quad (5)$$

Table S1: Scherrer Analysis for 9 nm hydride-terminated particles

Reflection	Angle (2θ)	FWHM (radians)	Calculated Crystallite Size (nm)
(111)	28.4	1.48	5.79
(220)	47.3	1.52	5.96
(311)	56.4	1.53	6.16

Table S2: Scherrer Analysis for 9 nm dodecyl-terminated particles

Reflection	Angle (2θ)	FWHM (radians)	Calculated Crystallite Size (nm)
(111)	28.4	1.38	6.22
(220)	47.3	1.42	6.38
(311)	56.2	1.59	5.92

Table S3: Scherrer Analysis for 6 nm dodecyl-terminated particles

Reflection	Angle (2θ)	FWHM (radians)	Calculated Crystallite Size (nm)
(111)	28.3	2.94	2.91
(220)	47.4	2.85	3.18

Table S4: Scherrer Analysis for 3 nm dodecyl-terminated particles

Reflection	Angle (2θ)	FWHM (radians)	Calculated Crystallite Size (nm)
(111)	27.4	6.37	1.34
(220)	47.6	5.44	1.67

Crystallite size calculated the Debeye-Scherrer Equation:

$$D = \frac{0.94\lambda}{B * Cos(\theta)}$$

Where:

λ = X-ray wavelength, 0.15418 nm used for Cu $K\alpha_1$ source

B = Full-width-at-half-maximum, shown in tables.

Table S5: Calculated Surface Coverage of SiNPs Averaged Over 55 Radical-Initiated Reactions

Time	3 nm	6 nm	9 nm
**2 h**	15.0 ± 7.4 %	17.1 ± 1.6 %	11.7 ± 2.9 %
**8 h**	16.8 ± 5.7 %	19.3 ± 1.4 %	16.1 ± 3.3 %
**24 h**	18.2 ± 4.6 %	27.4 ± 6.5 %	17.9 ± 6.0 %

Table S6: Weight Loss and Surface Coverage Values for TGA data presented

Sample	Weight Loss (%)	Calculated Surface Coverage (%)
**Fig 3a (2 h)**	6.18	6.8
**Fig 3a (8 h)**	14.23	17.0
**Fig 3a (24 h)**	15.00	18.1
**Fig 3b (2 h)**	3.18	3.4
**Fig 3b (8 h)**	3.98	4.3
**Fig 3b (24 h)**	21.05	27.4
**Fig S4 (65 °C)**	19.24	16.9
**Fig S4 (190 °C)**	33.37	35.6
**Fig S5 (Chloroform)**	9.75	4.3
**Fig S5 (Benzoyl Peroxide)**	30.84	17.7
**Fig S6 (No Initiator)**	9.29	7.3
**Fig S6 (AIBN)**	36.45	40.8
**Fig S7a (3 nm)**	27.68	15.2
**Fig S7a (6 nm)**	18.94	16.6
**Fig S7a (9 nm)**	12.50	14.7
**Fig S7b (3 nm)**	35.45	21.7
**Fig S7b (6 nm)**	33.2	35.3
**Fig S7b (9 nm)**	15.0	18.1
**Fig S8 (overetched)**	17.71	15.3
**Fig S8 (traditionally etched)**	37.72	43.1
**Fig S11 (hexene)**	11.78	5.3
**Fig S11 (octene)**	20.39	10.1
**Fig S11 (dodecene)**	35.35	21.7

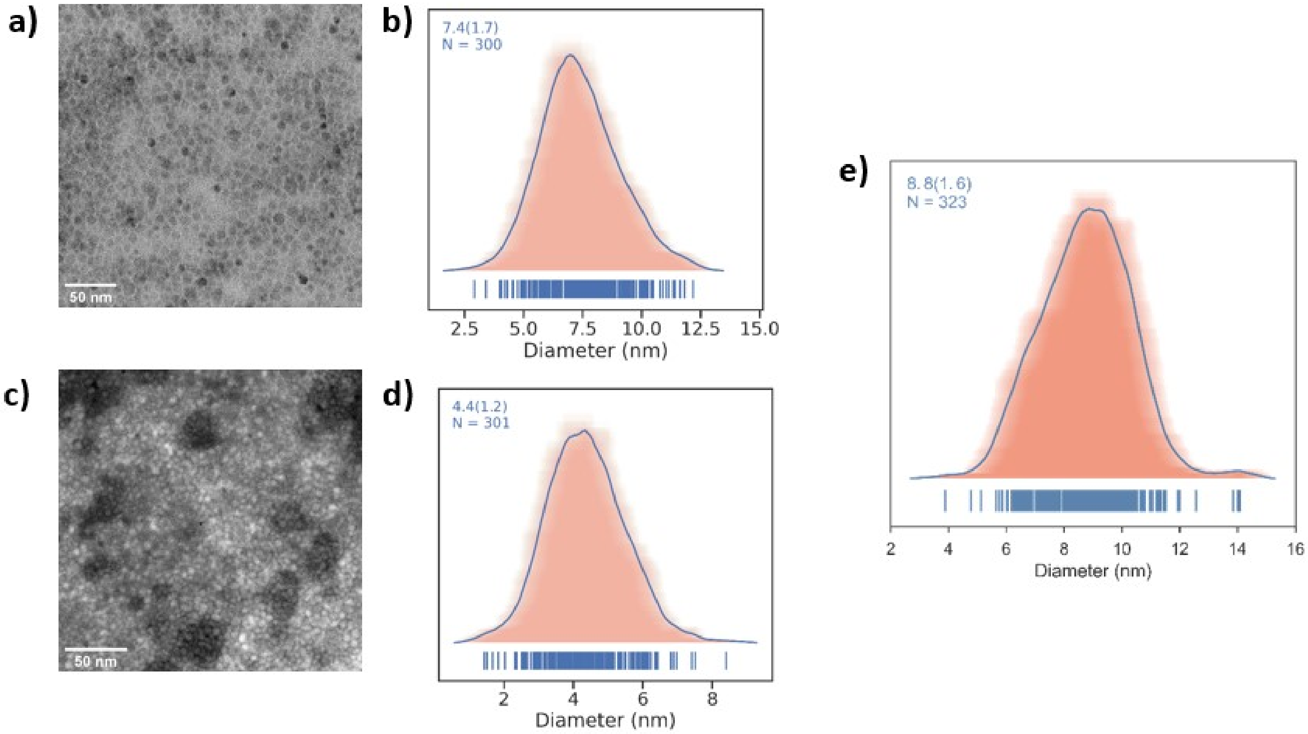

Figure S1: (a) Bright-Field TEM Image of 6 nm dodecyl-SiNPs and (c) Dark-Field TEM Image of 3 nm dodecyl-Si NPs, with corresponding average shifted histogram size distributions (b) and (d). e) Average shifted histogram for 9 nm particles, corresponding to Figure 1c.

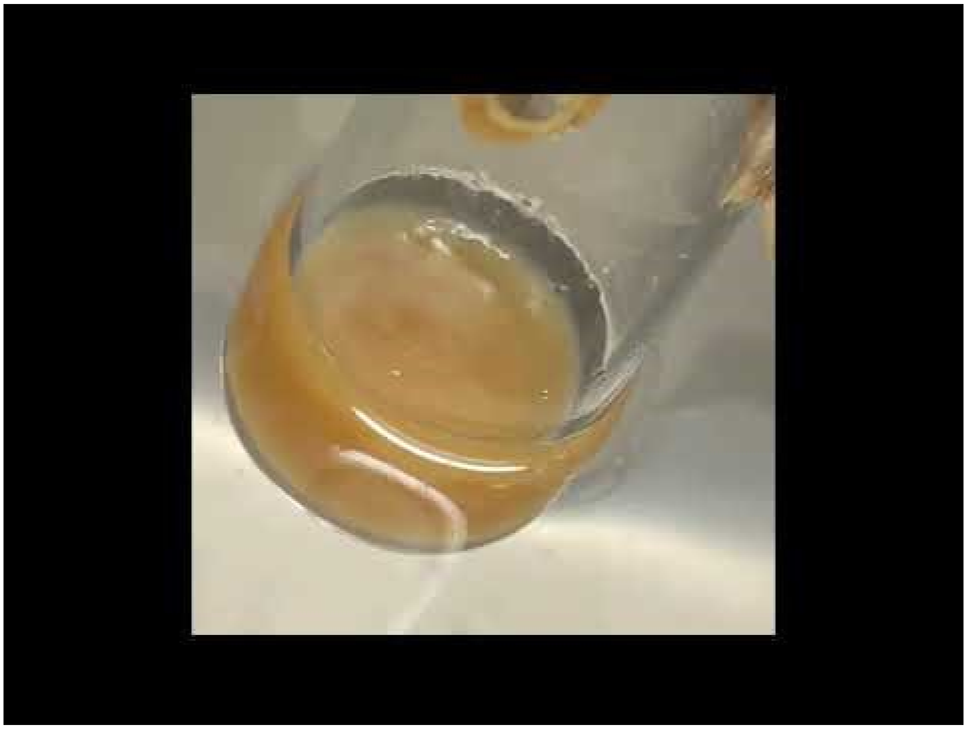

Figure S2: A 15x timelapse of the sonochemical hydrosilylation of 9 nm Si NPs with AIBN and 1-dodecene, showing the transition from opaque orange/brown to a transparent dark red solution.

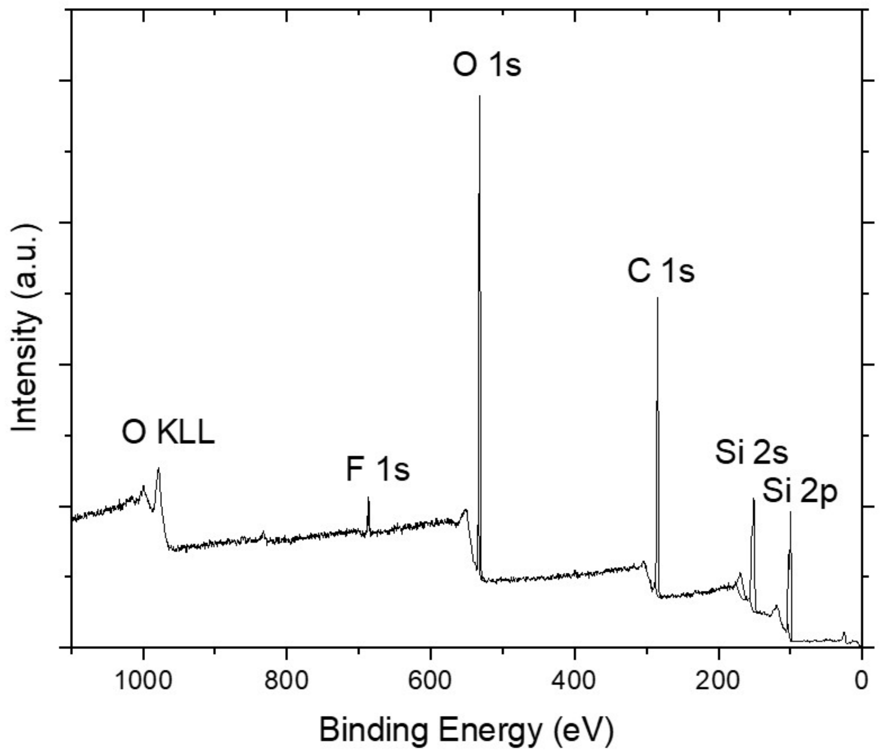

Figure S3: Survey XP Spectrum of 9 nm Si NPs sonicated with 1-dodecene for 24 h.

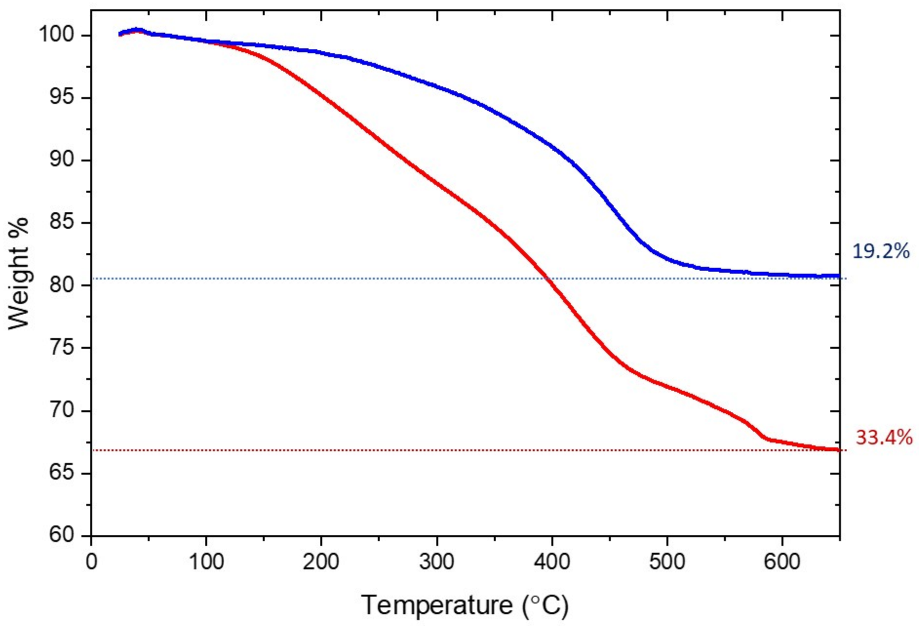

Figure S4: TGA Plot of 6 nm Si NPs functionalized with 1-dodecene and AIBN at 65 °C (blue) and at 195 °C with no initiator (red) after 24 h.

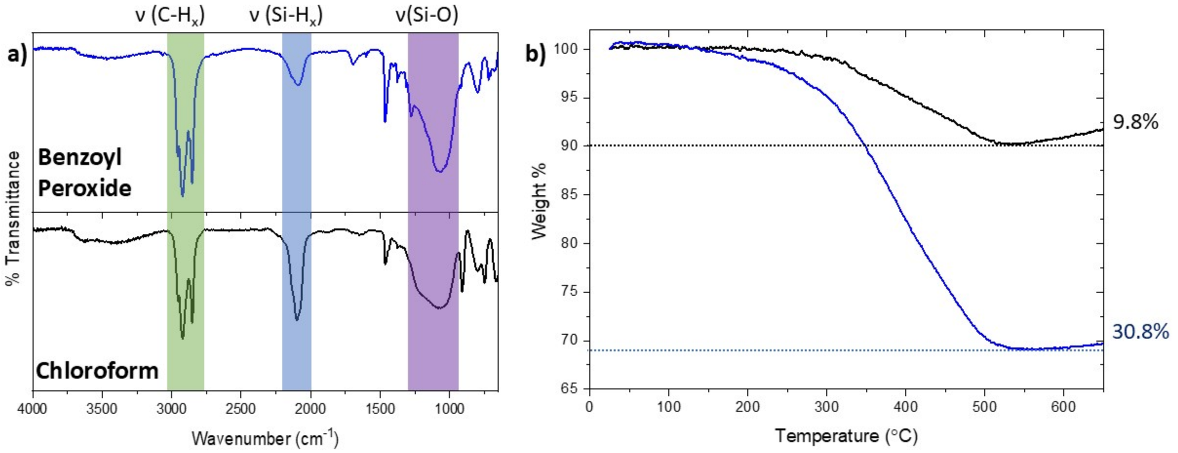

Figure S5: a) FTIR Spectra and b) TGA data of Benzoyl Peroxide initiated hydrosilylation (blue) and chloroform initiated hydrosilylation (black) using 1-dodecene and 3 nm SiNPs.

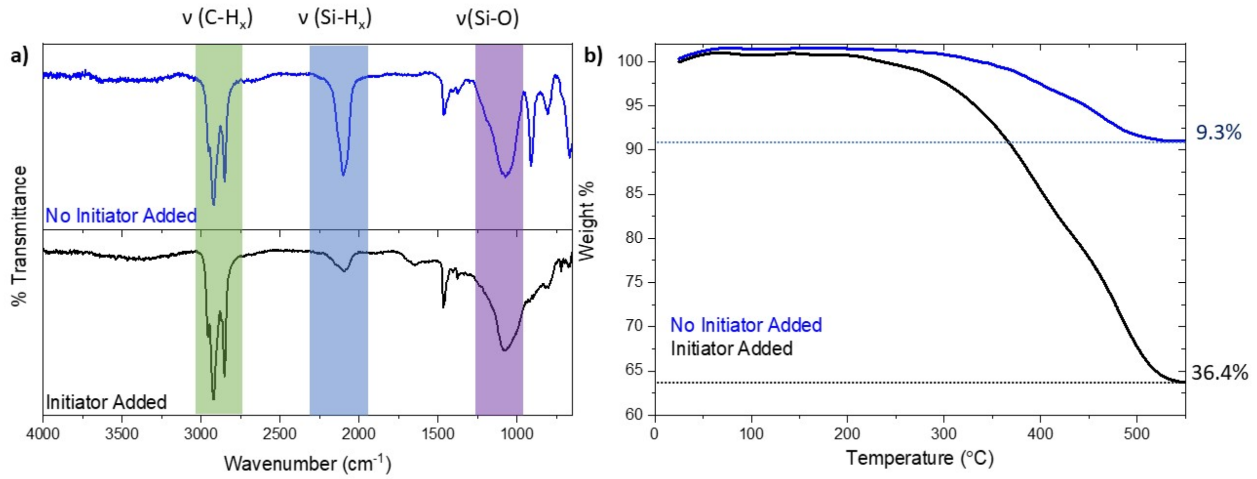

Figure S6: a) FTIR Spectra and b) TGA data of 6 nm Si NPs sonicated with 1-dodecene in 1,2-dichlorobenzene for 8 h, both in the presence and absence of AIBN.

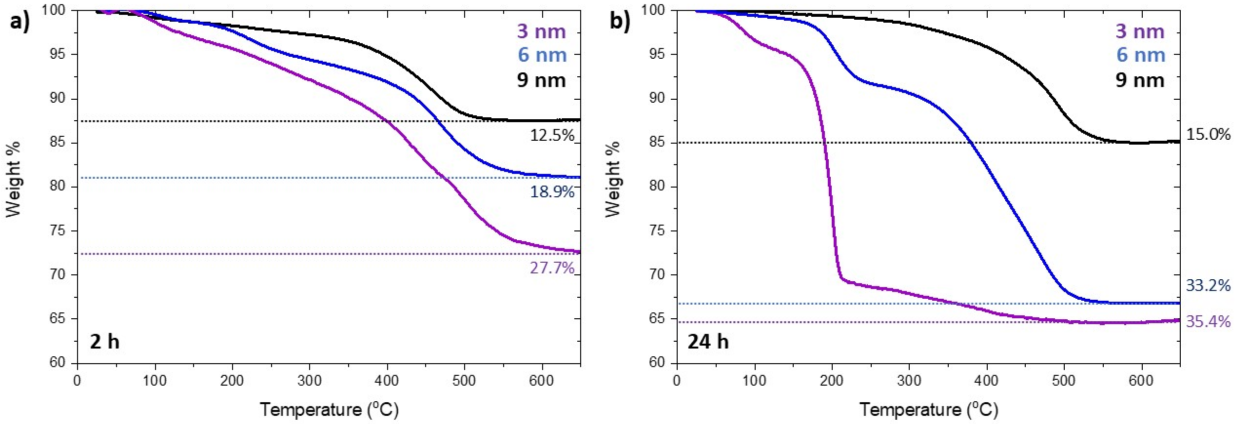

Figure S7: TGA plots of 3 (purple), 6 (blue) and 9 (black) nm Si NPs sonicated with Dodecene and AIBN for a) 2 h and b) 24 h.

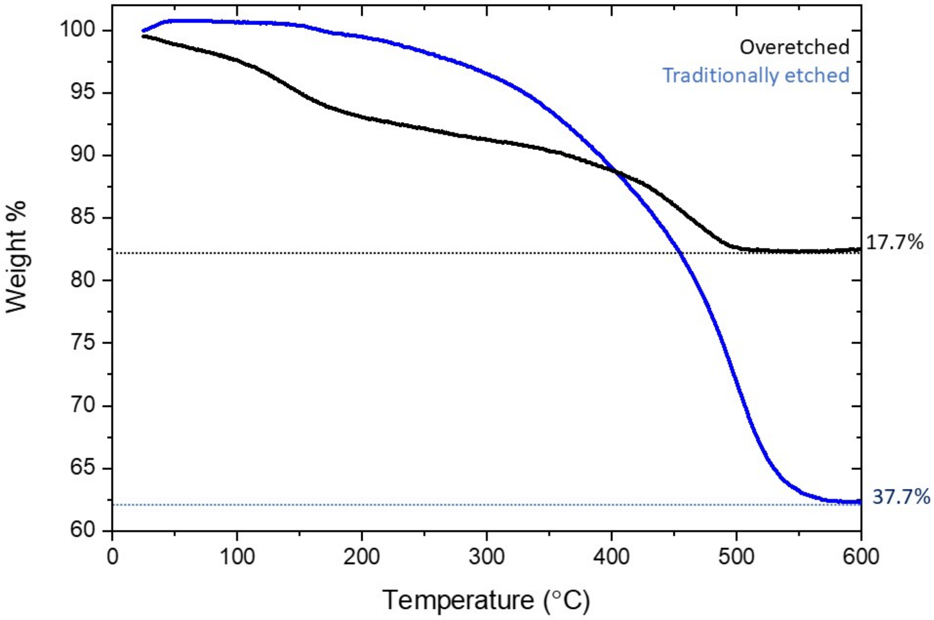

Figure S8: TGA plot of ca. 6 nm over-etched Si NPs (black) and traditionally etched 6 nm Si NPs (blue) sonicated with Dodecene for 24 h.

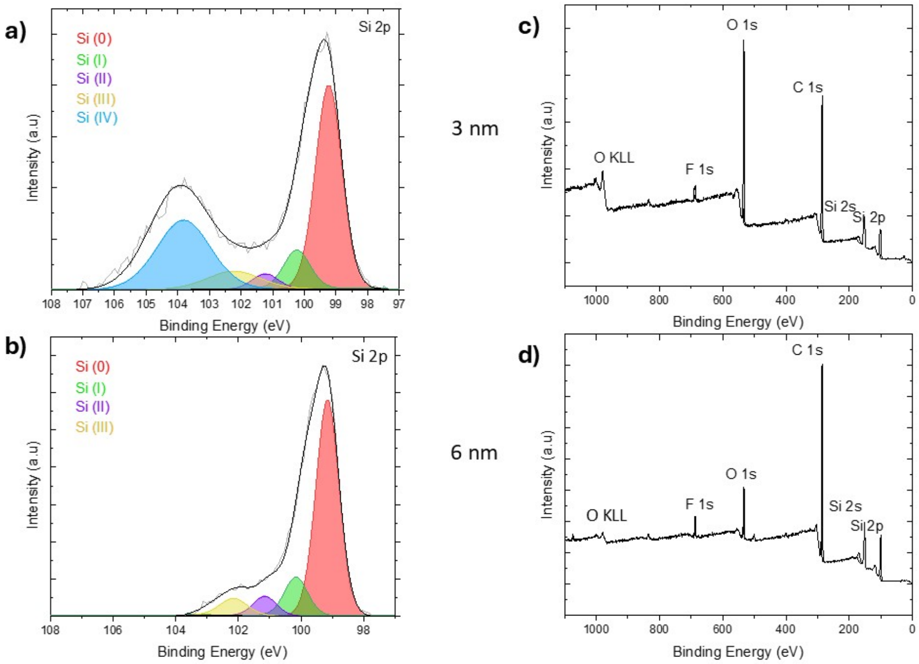

Figure S9: XP Spectra of a) 3 and b) 6 nm dodecyl-SiNPs sonicated for 24 h with AIBN, with corresponding survey spectra in c) and d)

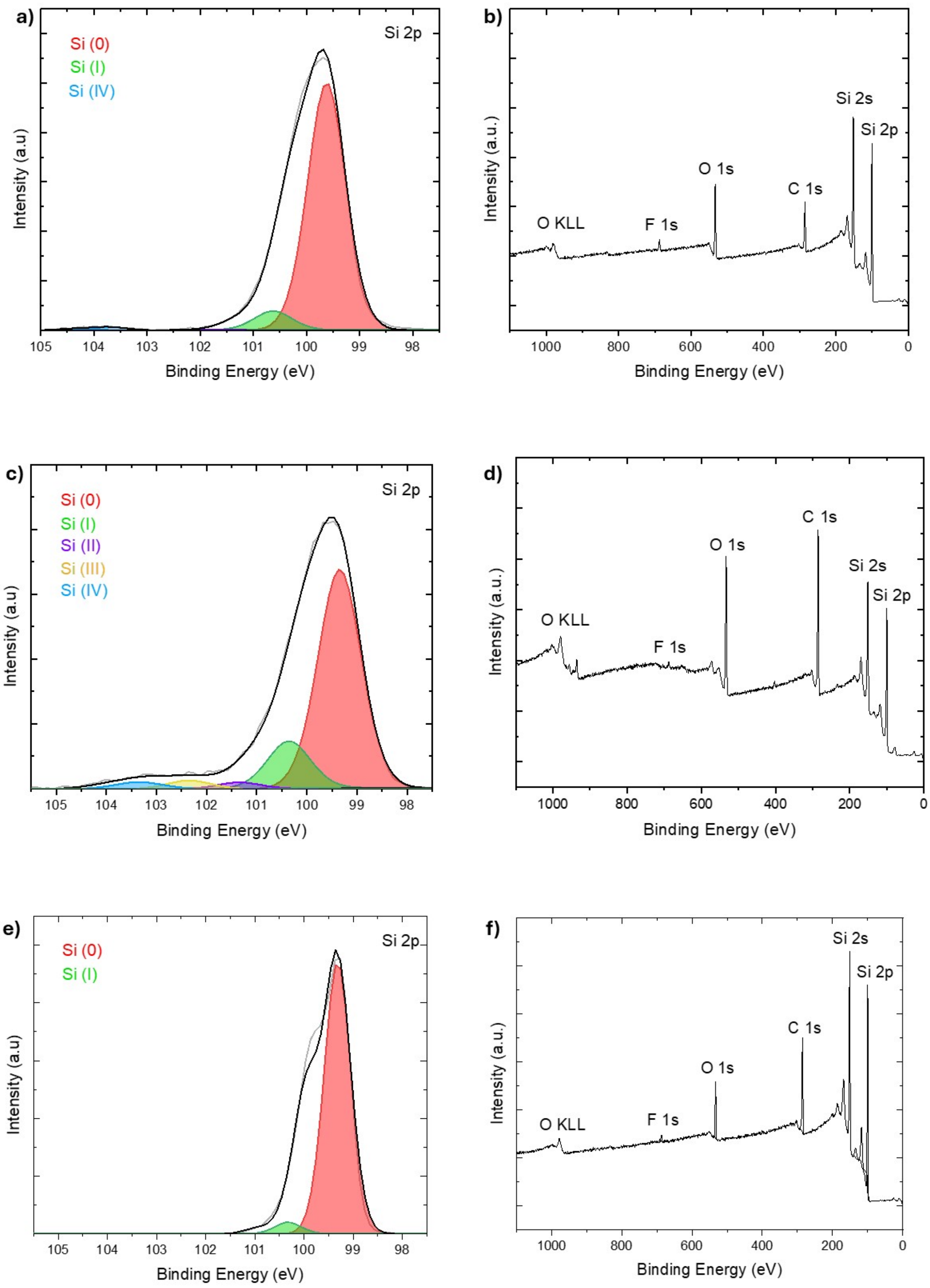

Figure S10: XP Spectra of H-SiNPs which are 3 (a), 6 (c), and 9 (e) nm in size, along with corresponding survey spectra (b, d, and f).

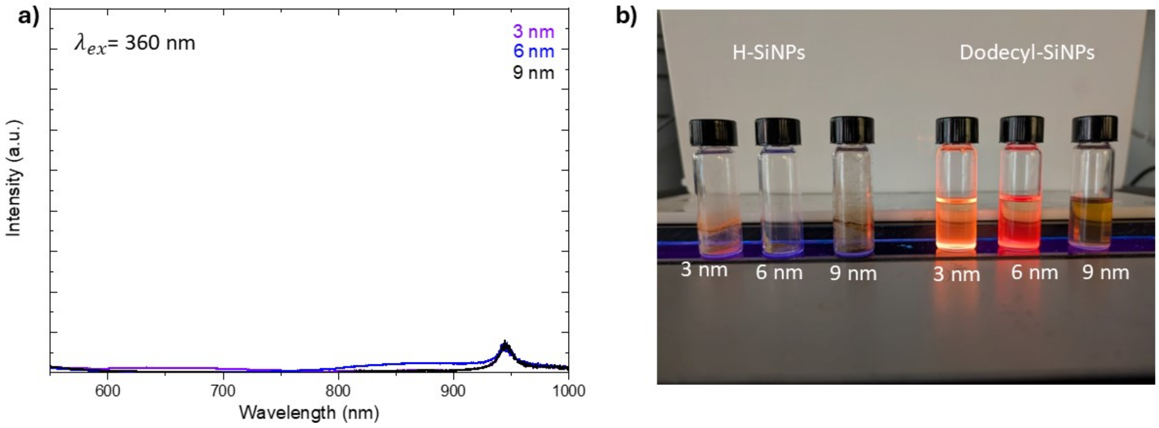

Figure S11: a) Photoluminescence spectra of 3, 6, and 9 nm H-SiNPs and b) photograph comparing photoluminescence response of H-SiNPs and Dodecyl SiNPs.

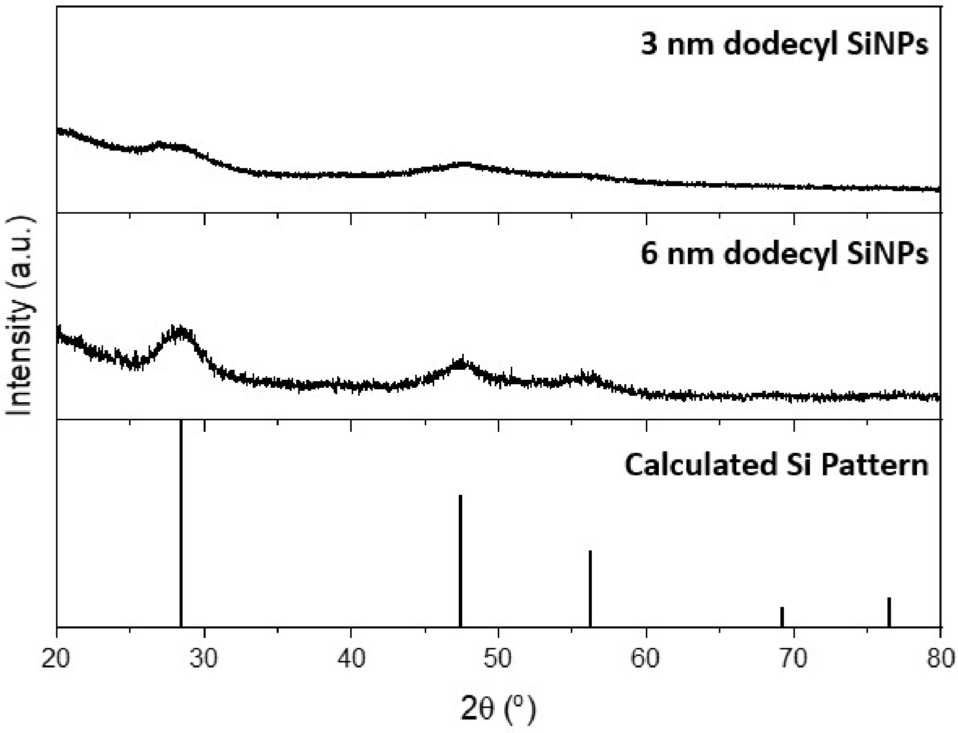

Figure S12: XRD Patterns obtained from 3 and 6 nm dodecyl-SiNPs sonicated for 24 h with AIBN.

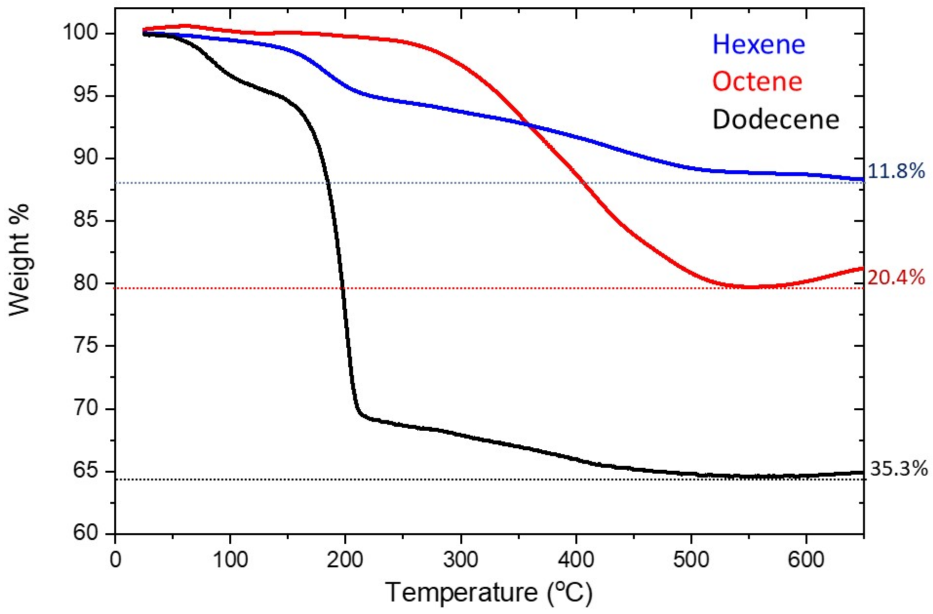

Figure S13: TGA Plot of sonochemically-functionalized 3 nm Si NPs obtained from 24 h reactions with AIBN and 1-hexene, 1-octene, or 1-dodecene, to yield surface coverages of 5, 10, and 22 %, respectively.
